# Childhood socioeconomic position and adult leisure-time physical activity: a systematic review

**DOI:** 10.1186/s12966-015-0250-0

**Published:** 2015-07-03

**Authors:** Ahmed Elhakeem, Rachel Cooper, David Bann, Rebecca Hardy

**Affiliations:** MRC Unit for Lifelong Health and Ageing at UCL, 33 Bedford Place, London, WC1B 5JU UK; Centre for Longitudinal Studies, UCL Institute of Education, 20 Bedford Way, London, WC1H 0AL UK

**Keywords:** Socioeconomic position, Socioeconomic inequalities, Physical activity, Exercise, Life course, Systematic review

## Abstract

Regular leisure-time physical activity (LTPA) benefits health and is thought to be less prevalent in lower socioeconomic groups. Evidence suggests that childhood socioeconomic circumstances can impact on adult health and behaviour however, it is unclear if this includes an influence on adult LTPA. This review tested the hypothesis that a lower childhood socioeconomic position (SEP) is associated with less frequent LTPA during adulthood. Studies were located through a systematic search of MEDLINE, Embase, PsycINFO, CINAHL and SPORTDiscus and by searching reference lists. Eligible studies were English-language publications testing the association between any indicator of childhood SEP and an LTPA outcome measured during adulthood. Forty-five papers from 36 studies, most of which were European, were included. In most samples, childhood SEP and LTPA were self-reported in midlife. Twenty-two studies found evidence to support the review’s hypothesis and thirteen studies found no association. Accounting for own adult SEP partly attenuated associations. There was more evidence of an association in British compared with Scandinavian cohorts and in women compared with men. Results did not vary by childhood SEP indicator or age at assessment of LTPA. This review found evidence of an association between less advantaged childhood SEP and less frequent LTPA during adulthood. Understanding how associations vary by gender and place could provide insights into underlying pathways.

**Electronic supplementary material:**

The online version of this article (doi:10.1186/s12966-015-0250-0) contains supplementary material, which is available to authorized users.

## Introduction

Physical activity (PA) is an important modifiable health behaviour implicated in the prevention of chronic disease and the promotion of health and mental well-being [1]. In addition, physical inactivity is a substantial public health burden [2]. Of the different domains of PA, leisure-time PA (LTPA) makes up the majority of time spent in moderate-to-vigorous intensity PA [3] and tends to be more strongly associated with favourable health outcomes [1, 4]. Evidence that LTPA levels have increased over time suggest that this domain of PA could be easier to modify than others [5] however, LTPA interventions generally report only small and short-term benefits [6].

Like many health-related outcomes, evidence from existing reviews indicates that LTPA is associated with contemporaneous socioeconomic circumstances [7–9]. Despite inconsistencies in the results as well as disagreement over whether certain indicators of socioeconomic position (SEP) appear to be more strongly related to LTPA than others [8, 9], the evidence suggests that less socioeconomically advantaged youth [7] and adults [8, 9] tend to participate less frequently in LTPA compared with their more advantaged peers.

In addition to more temporally adjacent associations between SEP and health, considerable evidence links childhood socioeconomic circumstances to adult health and behavioural outcomes [10]. These associations are typically of substantial magnitude and are not fully explained by the continuity of socioeconomic circumstances from childhood into adulthood [10]. It is plausible that adult LTPA mediates some of these associations or that adult LTPA itself exhibits early life socioeconomic origins. However, studies of the association between childhood SEP and adult LTPA have been inconsistent [11] and the literature has not been systematically reviewed.

A systematic review was carried out to test the hypothesis that a lower childhood SEP is associated with less frequent LTPA during adulthood. The extent to which associations were explained by the continuity of SEP from childhood into adulthood and between-study heterogeneity were explored.

## Methods

This systematic review, which was registered with the PROSPERO database (CRD42014007063) in January 2014, was carried out following the PRISMA guidelines [12] and a study protocol [13].

### Eligibility criteria

Included studies were those that tested the association between any recalled or prospectively ascertained indicator of childhood SEP (up to age 18 years) and an LTPA outcome measured from age 25. Studies were included if they reported results in English and published their findings in peer-reviewed journals. Observational studies using population-based samples were considered for inclusion.

Eligible indicators of childhood SEP were any resource and/or prestige-based measures of position within a societal structure [14] referring to participants’ early life (e.g. parental occupation/education, household amenities). Participants’ own education was not considered an eligible exposure despite its occasional use as an indicator of childhood SEP as it also captures the influence of adult resources [15].

Any PA performed during leisure-time was considered including sport, exercise and total LTPA [16]. The minimum age of 25 at measurement of LTPA, which equates the United Nations’ definition of adulthood [17], allows us to examine the long-term influences of childhood SEP and to inspect, in studies that account for own adult SEP, whether any associations are explained by the continuity of SEP from childhood to adulthood.

Reviews, unpublished literature, studies with non-LTPA outcomes (e.g. occupational PA only) or non-community based samples (e.g. hospital inpatients) were excluded.

### Search strategy

Embase (from 1974), MEDLINE (from 1946), PsycINFO (from 1806), CINAHL (from 1937) and SPORTDiscus (from 1985) were systematically searched using free-text synonym key-words (see Additional file 1) to locate all eligible studies available up to December 2014. Proximity and Boolean logic operators and truncation commands were used during the search [13]. Reference lists of included papers were searched to locate additional studies.

### Study selection

Results of the database searches were merged and duplicates removed. Abstracts were screened by two researchers (from AE, RC and RH) working independently and remaining full-texts of potentially eligible papers were double screened for inclusion. Disagreements were resolved through discussion between AE, RC and RH.

### Data extraction

The following data were extracted from all included papers (see Additional file 2): citation details, study details (e.g. design, setting, sample size), exposure and outcome details (e.g. type of indicators used and how and when these were ascertained), participant details (e.g. age, gender), statistical methods used, information on adjustment for potential confounding and mediating factors and lists of potentially eligible papers identified from reference lists. We extracted all statistics relating to the association of interest. A planned meta-analysis [13] was not attempted due to considerable heterogeneity in the reporting of results. All data were double extracted (by AE, RC, DB and RH) and discrepancies were resolved through discussion between these authors.

### Quality assessment

Study quality was assessed using the Newcastle-Ottawa Scale [18] which was modified [19] for this review. Quality was judged based on representativeness (of the study and source populations), adjustment for covariates, length of follow-up and methodology used to measure childhood SEP and adult LTPA (see Additional file 3). Quality scores were calculated as the average of two reviewers’ ratings with a potential range from 0 (lowest quality) to 9 (highest quality).

## Results

**Fig. 1:**
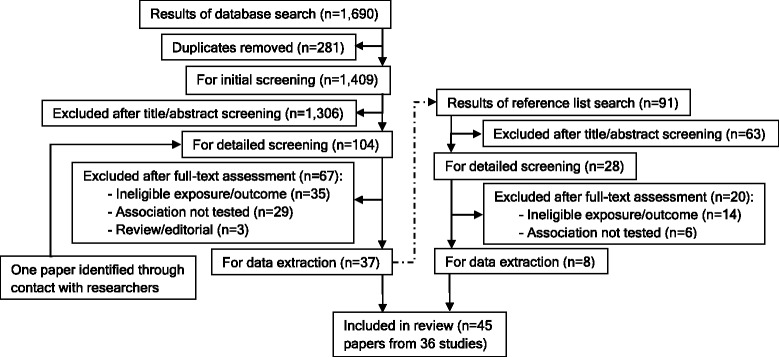
PRISMA study flow chart

A total of 1782 citations were identified. After initial screening and full-text assessments, 45 papers [20–64] reporting findings from 36 study samples underwent data extraction and were included for review (Fig. 1).

Characteristics of the included papers are presented in Table 1. Most (34/45) were based on European samples including 18 UK papers reporting on ten different study populations and 11 Scandinavian papers each from a unique study sample (four from Finland, three from Denmark and two from each of Norway and Sweden). Two papers each (from 2 studies) from Belgium and the Netherlands and one from Spain complete the European study settings. The remaining papers were eight US, one Australian, and two Chinese papers (the latter both reporting findings from the Guangzhou Bio-bank study (GBCS)). Some included papers did not address the review’s question as the primary association of interest and treated PA as a confounding or mediating factor but presented relevant associations [24, 27, 31, 36, 41, 52, 56, 57, 61].

**Table 1:** Characteristics of the included studies: arranged by region/country and from older to younger age at measurement of physical activity

-1st author (year)	-Description	-Childhood socioeconomic indicator/s^b^	-Physical activity measurement^c^	QA score^d^
-Country^a^ (birth year/s)	-Age at physical activity assessment	-How these were ascertained^b^	-Outcome/s of interest^c^	
-Study name	-Sample size (% female)			
-Johnson (2011) [20]	-Scottish birth cohort.	-PO (main occupation), PE, I&O (number of people per room, shared toilet facilities, whether indoor/outdoor toilet).	-Level of physical activities such as household chores, keep-fit, heavy exercise and sport.	3.5
-UK (1936)	-70 years.		-Physical activity six point score.	
-Lothian Birth Cohort 1936	-1091 (49.8 %).	-Recalled by SM at age 70.		
-Lawlor (2004) [21]	-Cross-section of women recruited from GP lists in 23 British towns.	-PO (longest held occupation).	-Hours per week spent on several types of domestic, recreational and sports activities.	4
-UK (1921–40)	-60–79 years.	-Recalled by SM at age 60–79.	-Physically inactive (<1 h/week. of moderate or vigorous physical activity).	
-British Women’s Heart & Health Study (BWHHS)	-3444 (100 %).			
-Hillsdon (2008) [22]	-Cross-section of women recruited from GP lists in 23 British towns.	-PO (longest held occupation), I&O (house with bathroom; hot water; shared bedroom, car access, and an index of all the above).	-Hours per week spent on several types of domestic, recreational and sports activities.	5
-UK (1921–40)	-60–79 years.		-Moderate to vigorous physical activity hours/week.	
-BWHHS	-4103 (100 %).	-Recalled by SM at age 60–79.		
-Watt (2009) [23]	-Cross-section of women recruited from GP lists in 23 British towns.	-PO (longest held occupation), I&O (house with bathroom; hot water; shared bedroom, car access, and an index of all the above).	-Hours per week spent on several types of domestic, recreational and sports activities.	4.5
-UK (1921–40)	-60–79 years.		-Low exercise (<2 h/week. of moderate or vigorous physical activity).	
-BWHHS	-3523 (100 %)	-Recalled by SM at age 60–79.		
-Ramsay (2009) [24]	-Cross-section of men recruited from GP lists in 24 British towns.	-PO (longest held occupation).	-Hours per week spent on several types of physical activities including walking, cycling and sports.	2.5
-UK (1920s-30s)	-52–74 years.	-Recalled by SM at age 52–74.		
-British Regional Heart Study (BRHS)	-5188 (0 %).		-Physically inactive (none or occasional physical activity).	
-Wannamethee (1996) [25]	-Cross-section of men recruited from GP lists in 24 British towns.	-PO (longest held occupation).	-No description (reference provided).	5
-UK (1920s-30s)	-40–59 years.	-Recalled by SM at age 52–74.	-Physically active.	
-BRHS	-2188 (0 %).			
-Stringhini (2013) [26]	-Cohort of civil servants employed in London.	-PO (main occupation).	-Hours per week spent on moderate and vigorous physical activities.	2
-UK (1930–53)	-40–59 years (phase 3).	-Recalled by SM at age 35–55.	-Physically inactive (≤1 h/week. of moderate and ≤1 h/week. of vigorous physical activity).	
-Whitehall II (WHII) Study	-6387 (28.5 %).			
-Heraclides (2008) [27]	-Cohort of civil servants employed in London.	-PO (main occupation).	-Hours per week spent on several types of domestic, recreational and sports activities.	3.5
-UK (1930–53)	-44–69 years (phase 5).	-Recalled by SM at age 35–55.	-Sedentary lifestyle (low quintile of MET score).	
-WHII Study	-4598 (26.8 %).			
-Brunner (1999) [28]	-Cohort of civil servants employed in London.	-PO (main occupation).	-Hours per week spent on several types of domestic, recreational and sports activities.	3.5
-UK (1930–53)	-35–55 years (phase 1).	-Recalled by SM at age 35–55.	-Physically inactive (no moderate or vigorous activities).	
-WHII study	-6980 (31.6 %).			
-Blane (1996) [29]	-Cross-section of men employed in 27 Scottish work places.	-PO (main occupation).	-Hours per week spent on exercise outside work including walking, gardening and golfing.	3
-UK (1908–37)	-35–64 years.	-Recalled by SM at age 35–64.		
-West of Scotland Collaborative Study	-5645 (0 %).		-Exercise hours/week.	
-Hart (1998) [30]	-Cross-section of men employed in 27 Scottish work places.	-PO (main occupation).	-Hours per week spent on exercise outside work including walking, gardening and golfing.	2.5
-UK (1908–37)	-35–64 years.	-Recalled by SM at age 35–64.		
-West of Scotland Collaborative Study	-5567 (0 %).		-Exercise hours/week.	
-Popham (2010) [31]	-Cross-section of Scottish residents.	-PO (when SM was aged 14)	-Frequency of several types sports and exercises during previous 4 weeks.	2.5
-UK (1949–68)	-35–54 Years.	-Recalled by SM at age 35–54.	-Sport and exercise (participated ≥ once in sport/exercise at moderate/high intensity for ≥15 min/day).	
-2003 Scottish Health Survey	-2770 (% unknown).			
-Hart (2008) [32]	-Cross-section of the 1970s Renfrew/Paisley Study offspring.	-PO.	-Frequency of daily activity and physical activity outside work.	5.5
-UK (1937–66)	-30–59 years.	-Reported by parents (SM was aged 6–39).	-No exercise (not very/at all active in daily activities and active for < once/week. or never outside of work).	
-Mid span family Study	-2338 (55.5 %).			
-Silverwood (2012) [33]	-British birth cohort.	-PO, PE.	-Latent classes for a) walking during work and pleasure b) cycling during work and pleasure and c) LTPA.	5.5
-UK (1946)	-36–53 years.	-Reported by parents (SM was aged 4 and 6).		
-MRC National Survey of Health and Development (NSHD)	-3847 (49.6 %).		-LTPA (low, gardening & DIY, sports), walking, cycling (low, high).	
-Kuh & Cooper (1992) [34]	-British birth cohort.	-PO, PE.	-Frequency of several types of sports and recreational activities during previous month.	7
-UK (1946)	-36 years.	-Reported by parents (SM was aged 4 and 6).	-High participation in sport and recreational activities.	
-MRC NSHD	-2144 (50.3 %).			
-Pinto Pereira (2014) [35]	-British birth cohort.	-PO, PE, I&O (index of household amenities: availability of bathroom, indoor lavatory and hot water).	-Frequency of LTPA such as swimming, going for walks.	6
-UK (1958)	-33, 42, 50 years.		-Low LTPA (< once/week).	
-National Child Development Study 1958 (NCDS)	-12,776 had ≥ one measure of LTPA.	-Reported by parents at SM’s birth and when aged 7, 11 and 16.		
-Cheng & Furnham (2013) [36]	-British birth cohort.	-PO (current or last held occupation).	-Frequency of physical exercise.	3
-UK (1958)	-50 years.	-Reported by parent at SM’s birth.	-Exercise score (6-point scale).	
-NCDS	-5921 (49.4 %).			
-Juneau (2014) [37]	-British birth cohort.	-PO.	-Frequency of LTPA during the previous 8 weeks.	5
-UK (1970)	-34 years.	-Reported by parents at SM’s birth and when aged 5 and 10 years.	-Estimated LTPA energy expenditure.	
-1970 British Cohort Study	-9624 (52.2 %).			
-Osler (2008) [38]	-Danish birth cohort of men from Copenhagen.	-PO.	-Frequency of walking, running, cycling and other activities.	6.5
-Denmark (1953)	-51 years.	-Extracted from birth records.	-Sedentary leisure activity (mainly reading, watching TV or having other sedentary activities during leisure).	
-Metropolit Birth Cohort	-6292 (0 %).			
-Lynch (1997) [39]	-Cross-section of men from Eastern Finland.	-I&O (index of PO, PE, whether family perceived as wealthy, whether family lived on a farm and size of farm).	-Energy expended in LTPA during the previous 12 months, e.g. jogging, swimming, cycling, skiing.	2.5
-Finland (1920s-40s)	-42–60 years.			
-Kuopio Ischaemic Heart Disease Risk Factor Study	-2682 (0 %).	-Recalled by SM at age 42–60.	-(i) No conditioning activities, (ii) low quartile of conditioning activities.	
-Kvaavik (2011) [40]	-Follow-up of Oslo students invited to a health education intervention.	-PE.	-‘How often do you exercise for at least half an hour to the extent that you sweat and/or are short of breath?’	6
-Norway (1964–8)	-25, 33, 40 years.	-Reported by parents (SM aged 11–16).		
-Oslo Youth Study	-240, 329, 407.		-LTPA (twice/week).	
-Jørgensen (2013) [41]	-Cohort of Danish women employed as social and health care assistants.	-PO (when SM was aged 14).	-Hours per week spent on LTPA.	0.5
-Denmark (≈1971)	-35.4 years (SD = 10.5)	-Recalled by SM at age 35.4.	- Low LTPA (<4 h/week).	
-Danish Health Care Worker Cohort	-1661 (100 %).			
-Barnekow-Bergkvist (1998) [42]	-Follow-up of Swedish students.	-PO.	-Hours per week spent on LTPA (includes sports, walking, and cycling) in the previous 12 months.	3
-Sweden (1958)	-34 years.	-Recalled by SM at age 34.		
			-LTPA MET hours/week.	
	-278 (43.5 %).			
-Tammelin (2003) [43]	-Northern Finland birth cohort.	-PO.	-Frequency of light and brisk LTPA.	5.5
-Finland (1966)	-31 years.	-Reported when SM aged 14.	-Physically inactive (brisk LTPA < once/week. and light LTPA <4 times/week).	
-North Finland Birth Cohort 1966	-7794 (53 %).			
-Makinen (2009) [44]	-Regionally stratified cross-section of Finnish adults.	-PO, PE, I&O (long-term financial problems in family, regular parental unemployment–both before age 16).	-How much do you exercise and strain yourself physically in leisure time?’	3.5
-Finland (1970 & older)	-30+ years.		-Inactive (read, watch TV or do other activities that do not strain me physically); moderately active (walk, cycle or move in other ways for at least 4 h/week).	
-Health 2000 Survey	-7112 (55.4 %).	-Recalled by SM at age 30+.		
-Wichstrøm (2013) [45]	-Follow-up of students from 67 Norwegian schools.	-PO.	-Hours spent on physical exercise during the previous week.	4.5
-Norway (1973–80)	-25–32 years.	-Reported by SM at age 12–19.	-LTPA hours/week.	
-Young in Norway Study	-2890–2923.			
-Leino (1999) [46]	-Follow-up of Finnish children and adolescents.	-PE.	-Frequency and duration of exercise used to form an LTPA index.	4
-Finland (1962–71)	-21–30 years.	-Reported by SM at age 9–18.	-Physically inactive (≤25th percentile of LTPA index, range = 0–52).	
-Cardiovascular Risk in Young Finns Study	-432 (53.7 %).			
-Osler (2001) [47]	-Follow-up of CCHS offspring aged 6–18 at baseline.	-PE.	-Current level of participation in LTPA and whether active in sports.	7
-Denmark (1961–73)	-19–31 years.	-Reported by parents (SM aged 6–18).	-Low LTPA (mostly sitting or light activity for ≥4 h/week. and not active in sports).	
-Offspring of Copenhagen City Heart Study (CCHS)	-317 (48.9 %).			
-Peck (1994) [48]	-Cross-section of employed Swedes.	-PO (during SM’s childhood).	-Regular LTPA (no description).	1.5
-Sweden (1900s-60s)	-16–74 years.	-Recalled by SM at age 16–74.	-No regular LTPA.	
	-12,695 (50.4 %).			
-Regidor (2004) [49]	-Cross-section of an older Spanish population.	-PO.	-Type of physical activity done in spare time or at any time if retired/unemployed.	4.5
-Spain (1940 & older)	-60+ years.	-Recalled by SM at age 60+.	-Physically inactive (only report sedentary activities e.g. reading, watching TV).	
	-3658 (54.6 %).			
-Beunen (2004) [50]	-27-year follow-up of Flemish speaking adolescent Belgian boys.	-PO, PE, I&O (degree of urbanisation).	-Frequency of sports, other leisure-time activities and accelerometer counts of daily physical activity.	5.5
-Belgium (1956)	-40 years.	-Reported by SM at age 14–18.		
-Leuven Longitudinal Study of Flemish Boys	-166 (0 %).		-Sport, leisure-time, & counts indices.	
-Scheerder (2006) [51]	-20-year follow up of Flemish speaking adolescent Belgian girls.	-I&O (index of PO and PE).	-Hours per week spent on sports during the previous year.	6
-Belgium (1961–7)	-32–41 years.	-Reported by SM at age 12–18.	-Level of sports participation (hours/week./year).	
-Leuven Longitudinal Study of Flemish Girls	-257 (100 %).			
-Kamphuis (2013) [52]	-Cross-section of men living in or near Eindhoven.	-PO (when SM was aged 12).	-Hours per week spent on transport, leisure-time and sports related activities.	2
-Netherlands (1916–51)	-40–75 years.	-Recalled by SM at age 40–75.	-Physically active (≥3.5 h/week. of sports and transport or leisure-time physical activity).	
-GLOBE Study	-4894 (0 %)			
-van de Mheen (1998) [53]	-Cross-section of adults living in or near Eindhoven.	-PO (when SM was aged 11).	-Leisure-time physical exercise (no description).	3.5
-Netherlands (1910s-60s)	-25–74 years.	-Recalled by SM at age 25–74.	-Frequent LTPA, and no LTPA.	
-Longitudinal Study on Socio-Economic Health Differences	-13,854 (% unknown).			
-Pudrovska (2013) [54]	-Long-term follow-up of high school graduates from Wisconsin.	-I&O (index of PO, PE, family income, father’s occupational income and father’s occupational education).	-Hours per month spent on light (e.g. walking, gardening, golfing) and vigorous (e.g. aerobics, jogging, swimming) physical activities.	6
-US (1939–40)	-65 years.			
-Wisconsin Longitudinal Study	-5778 (54.7 %).	-Reported when SM was aged 17–18.	-Physical activity index.	
-Wray (2005) [55]	-Follow-up of middle aged and older US adults.	-PE.	-Whether or not SM is a vigorous exerciser. Includes heavy housework, cycling, aerobics, running, jogging, swimming and physical labour at work.	5
-US (1941 & older).	-51–61 years (HRS); 70+ years (AHEAD).	-Recalled by SM at age 51–61 (HRS) and 70+ (AHEAD).		
-Health & Retirement Study (HRS); Study of Asset & Health Dynamics (AHEAD)	-HRS: 6106 (57 %); AHEAD: 3636 (63 %).		-Low physical activity (not exercising ≥3 times/week).	
-Bowen (2010) [56]	-Cohort of middle aged and older US adults.	-PO (main occupation), PE.	-Whether or not SM is a vigorous exerciser. Includes heavy housework, cycling, aerobics, running, jogging, swimming and physical labour at work.	3
-US (1941 & older)	-51+ years.	-Recalled by SM at age 51+.		
-HRS merged with AHEAD and two other cohorts	-18,465 (60 %).		-Vigorous exercisers (≥3 times/week).	
-Carroll (2011) [57]	-Cross-section of Pennsylvanian adults recruited to a Hepatitis B vaccination project.	-I&O (index for every 2 years of childhood: whether parents owned home, number of a) bathrooms, b) people living in the home and c) vehicles owned).	-Paffenbarger physical activity questionnaire (no description).	1
-US (1950s-70s)			-Physical activity kilocalories expended per week.	
-Vaccination Immunity Project	-40–60 years.	-Recalled by SM at age 40–60.		
	-153 (59.8 %).			
-Frank (2003) [58]	-Cross-section of women physicians born in the US.	-PE.	-Exercise (no description).	0.5
-US (1930–50)	-30–70 years.	-Recalled by SM at age 30–70.	-Exercising ≥30 min on 3 times per week.	
-Women Physician Health Study	-2884 (100 %).			
-Tsenkova (2014) [59]	-Cross-section of US adults who participated in a biomarkers study.	-I&O (index of PE, childhood welfare status and financial level growing up).	-‘How often do you engage in vigorous physical activity long enough to work up a sweat (e.g. running/heavy lifting)?’	3
-US (1921–70)	-25–74 years.	-Recalled by SM at age 25–74.		
-Midlife in the US Study	-895 (54.6 %)		-Exercise sessions per month.	
-Kern (2010) [60]	-Long-term follow-up of Californian children with high IQ.	-I&O (index of PO and PE).	-Avocational activities and hobbies including sport, gardening, music, art, writing, photography.	4.5
-US (1910s)	-25–61 years.	-Reported by parents (SM was aged 11).		
-Terman Life Cycle Study	-1114 (50 %).		-Average physical activity METs.	
-Phillips (2009) [61]	-Cross-section of Pennsylvanian adults without serious illnesses.	-PE.	-Paffenbarger physical activity questionnaire (no description).	2.5
-US (1940s-70s)	-30–54 years.	-Recalled by SM at age 30–54.	-Physical activity kilocalories expended per week.	
-Adult Health and Behaviour Project	-811 (51.4 %).			
-Schooling (2007) [62]	-Cross-section of Guangzhou community club members.	-I&O (number of parental possessions from a watch, sewing machine and bicycle during SM’s childhood).	-IPAQ used (no description).	3
-China (1955 & older)	-50+ years.		-Inactive, minimally active, and HEPA (vigorous activity ≥3 days/week. at ≥1500 MET minutes/week, or activity 7 days/week. at ≥3000 MET minutes/week).	
-Guangzhou Bio-bank Cohort Study (GBCS)		-Recalled by SM at age 50+.		
	-9748 (71.9 %).			
-Elwell-Sutton (2011) [63]	-Cross-section of Guangzhou community club members.	-I&O (number of parental possessions from a watch, sewing machine and bicycle during SM’s childhood).	-IPAQ used (no description).	3
-China (1955 & older)	-50+ years.		-Inactive, minimally active, and HEPA (vigorous activity ≥3 days/week. at ≥1500 MET minutes/week, or activity 7 days/week. at ≥3000 MET minutes/week).	
-GBCS		-Recalled by SM at age 50+.		
	-20,086 (73.2 %).			
-Gall (2010) [64]	-20-year follow-up of the Australian Schools Health & Fitness Survey.	-PE.	-Whether or not SM participated in ≥3 h of moderate/vigorous LTPA per week.	4.5
-Australia (1970s)	-26–36 years.	-Recalled by SM at age 26–36.	-LTPA (≥3 h/week).	
-Childhood Determinants of Adult Health Study	-1973 (52.8 %).			

^a^ *UK* United Kingdom, *US* United States, Nordic group of countries (Norway, Sweden, Finland and Denmark) considered as one region

^b^ *PO* Parental occupation (usually based on father’s occupation, more detail can be found in brackets if provided in the paper), *PE* Parental education (years and/or level), *I&O* Indices and other measures of childhood socioeconomic position (SEP), includes (i) indices combining different indicators of childhood SEP and (ii) single measures which are distinct from parental occupation and education, *SM* Study member

^c^ *LTPA* Leisure-time Physical Activity, *METs* Metabolic equivalents, *IPAQ* International Physical activity Questionnaire, *HEPA* Health enhancing physical activity: acronym used in the two GBCS papers [62, 63]

^d^ *QA score* Quality assessment score (average of two assessor’s scores possible values are 0–9)

Study sample sizes varied from 112 to 20,086 and mostly comprised adults whose LTPA was ascertained in midlife. Birth years were from the early 1900s to 1980 and participants were mostly drawn from the general population though four study populations were sampled from occupational settings [26–30, 41, 58]. The majority of included papers (*n* = 34) had a medium quality score (3 to 5) although the range was considerable (0.5 to 7).

Twenty-nine papers (22 studies) relied on participants recalling childhood SEP and in sixteen (14 studies) it was ascertained prospectively. For this review, different measures of childhood SEP were grouped into a) parental occupation, b) parental education and c) indices (combining >1 measure) and other indicators of childhood SEP (e.g. car access). Eight papers (7 studies) [20, 22, 23, 33, 35, 44, 50, 56] present results from at least two of the above and four (4 studies) [20, 35, 44, 50] report associations for each group of childhood SEP measures. PA was measured by self-report with the exception of Beunen et al. [50] who present both accelerometer and self-reported outcomes. Questions used to collect PA ranged from single-items [40, 44, 59] to detailed questionnaires [34]. Not all outcomes were LTPA-specific as three papers present outcomes conflating work-related activity and LTPA [33, 55, 56] and some provide no description of what PA domains are included in their outcome (but which are assumed to include LTPA) [25, 57, 61].

### Association between childhood SEP and adult LTPA

Results were presented as prevalence of LTPA by childhood SEP group, correlation between SEP and LTPA or regression coefficients from statistical models. Overall, results supported the hypothesis that a lower childhood SEP is associated with less frequent adult LTPA however, several null findings were reported. Two studies found evidence of an association between lower childhood SEP and higher adult PA [33, 62]. Results are summarised by three groups of childhood SEP indicators (Tables 2, 3, 4).

**Table 2:** Results of studies testing the association between parental occupational class and leisure-time physical activity (LTPA) in adults

-1st author (year)	How results presented and interpretation^b^	Correlations coefficient/difference in prevalence^c^	Estimates from statistical modelling^c^	Adjustments^d^
-Country; study name				
-Sample size^a^; age				
-Johnson (2011) [20]	Correlation and regression coefficients for a 6-point LTPA score and parental occupation (RGSC 1951: I, II, IIIN, IIIM, IV, V) (per unit change from high to low occupational class in regression model).	*r* = −0.06 (+, *p* = 0.05)		None
-UK; Lothian Birth Cohort 1936			β = −0.01 (ns)	Education, own occupational class, other childhood SEP, IQ & more
-1091; 70+ Yrs.				
-Lawlor (2004) [21]	Prevalence of physical inactivity in six parental occupational groups (RGSC 1980: I, II, IIIN, IIIM, IV, V) and odds of physical inactivity per unit increase from high to low occupational class.	I-IV = −11.4 % {−13.6; −6.4} (+)		None
-UK; British Women’s Heart & Health Study (BWHHS)				
-3444^♀^; 60–79 years.			OR = 1.17 {1.08; 1.26} (+)	Age
			OR = 1.15 {1.06; 1.25} (+)	Age, own occupational class
-Hillsdon (2008) [22]	Prevalence of manual parental occupational class (RGSC 1980) in four groups of physical activity hours/week.	% manual occupations:		None
-UK; BWHHS		≥3–0 h/week. = −7.4 %		
-4103^♀^; 60–79 years.		{−6.1; −8.6} (+, *p* < 0.001)		
-Watt (2009) [23]	Percentage difference in low exercise between manual (M) and non-manual (NM) parental occupations (RGSC 1980).	NM-M = −6.7 % {−2.5; −10.9} (+, *p* < 0.01)		None
-UK; BWHHS				
-3523^♀^; 60–79 years.				
-Ramsay (2009) [24]	Prevalence of physical inactivity in manual (M) and non-manual (NM) parental occupations (RGSC 1980).	NM-M = −48 % (+, *p* = 0.05)		None
-UK; British Regional Heart Study (BRHS)				
-5188^♂^; 52–73 years.				
-Wannamethee (1996) [25]	Prevalence of physical activity in manual (M) and non-manual (NM) parental occupations (RGSC 1980).	NM-M = 8 % (+, *p* < 0.0001)		None
-UK; BRHS		NM-M = 2.4 % (ns)		Age, own occupational class
-5516^♂^; 40–59 years.				
-Stringhini (2013) [26]	Odds of physical inactivity in the lowest compared to the highest tertile of parental occupation (RGSC 1980).		OR = 1.37 {1.14; 1.65} (+, *p* < 0.05)	Age, sex, ethnicity, CHD, stroke, cancer, hypertension, family history of diabetes
-UK; Whitehall II (WHII) Study				
-6387; 40–59 years.				
-Heraclides (2008) [27]	Prevalence of physical inactivity in manual (M) and non-manual (NM) parental occupations (RGSC 1980).	NM-M:		None
-UK; WHII Study		♂ = 1.9 % (ns)		
-4598; 44–69 years.		♀ = 1.3 % (ns)		
				sd
-Brunner (1999) [28]	Prevalence of physical inactivity in four parental occupational groups (RGSC 1980: I/II, IIIN, IIIM, IV/V).	I-IV (♂) = −4.8 % (+, *p* = 0.01)		Age
-UK; WHII Study		I-IV (♀) = −7.9 % (+, *p* = 0.02)		
-6980; 35–55 years.		I-IV (♂) = −2.6 % (ns)		Age, own occupational class
		I-IV (♀) = −2.9 % (ns)		
-Blane (1996) [29]	Prevalence and regression coefficients for mean exercise hours/week. by four parental occupational groups (RGSC 1966: I/II, IIIN, IIIM, IV/V).	I/II-IV/V = 0.7 h/week. {SE: I/II =0.13; IV/V =0.16}		Age
-UK; West of Scotland Collaborative Study				
-5646^♂^; 35–64 years.			β = −0.16 {−0.32; 0.01} (ns)	Age
-Hart (1998) [30]	Prevalence of exercise hours/week. in four groups of parental and own occupations (RGSC 1966: 1. stable non-manual 2. moved up 3. moved down 4. stable manual).	1–4 = 0.5 h/week. (+, *p* = 0.002)		Age
-UK; West of Scotland Collaborative Study				
-5567^♂^; 35–64 years.				
-Popham (2010) [31]	Prevalence of sport & exercise in four parental occupational groups (RGSC: I/II, IIIN, IIIM, IV/V).	I/II-IV/V = 18.6 % {17.7; 19.6} (+)		Age, sex
-UK; 2003 Scottish Health Survey				
-2770; 35–54 years.				
-Hart (2008) [32]	Prevalence of no exercise in manual (M) and non-manual (NM) parental occupations (RGSC 1966) and odds of no exercise per unit increase (1–6) from low to high parental occupational class.	NM-M:		None
-UK; Mid span Family Study		♂ = 3.7 % (ns)		
-2338; 30–59 years.		♀ = −3.0 % (ns)		
			Odds Ratios:	Age
			♂ = 1.03 (0.91; 1.16) (ns)	
			♀ = 1.09 (0.98; 1.21) (ns)	
-Silverwood (2012) [33]	Prevalence of LTPA (low; gardening; sport & leisure), walking and cycling during work & for pleasure (high, low) in four parental occupational groups (RGSC 1970: I/II, IIIN, IIIM, IV/V).	I/II-IV/V: LTPA (sports & leisure):		None
-UK; MRC National Survey of Health and Development (NSHD)		♂ = 12.2 % (+, *p* < 0.001)		
		♀ = 17.9 % (+, *p* < 0.001)		
-> 3300; 31–53 years.		I/II-IV/V: Walking (high):		
		♂ = −17.6 % (−, *p* < 0.001)		
		♀ = −6.6 % (−, *p* = 0.002)		
-Kuh & Cooper (1992) [34]	Prevalence of most active in sports & recreational activities in four parental occupational groups (RGSC 1970: I/II, IIIN, IIIM, IV/V).	I/II-IV/V:		None
-UK; MRC NSHD		♂ = 9.1 % (ns)		
-2977; 36 years.		♀ = 21.4 % (+, *p* < 0.001)		
-Pinto Pereira (2014) [35]	Odds of low LTPA per unit increase from high to low parental occupational class (RGSC 1951: I/II, IIIN, IIIM, IV/V).		Odds Ratios:	None
-UK; National Child Development Study 1958 (NCDS)			Age 33 = 1.12 {1.07; 1.16} (+)	
-12,776 had ≥ one measure of LTPA; 33, 42, 50 year.			Age 42 = 1.16 {1.11; 1.20} (+)	
			Age 50 = 1.23 {1.17; 1.29} (+)	
			Age 33 = 1.06 {1.01; 1.11} (+)	Sex
			Age 42 = 1.10 {1.05; 1.15} (+)	
			Age 50 = 1.13 {1.07; 1.19} (+)	
			Age 33 = 1.01 {0.97; 1.06} (ns)	Sex, parental education, aptitude, household amenities, cognition, lifestyle factors age 16, & more
			Age 42 = 1.05 {1.002; 1.10} (+)	
			Age 50 = 1.09 {1.03; 1.15} (+)	
			Age 33 = 1.00 (0.95; 1.05) (ns)	As above plus own education, own social class, BMI, mental health, number of children in the household, limiting illness
			Age 42 = 1.04 (0.99; 1.09) (ns)	
			Age 50 = 1.07 (1.01; 1.13) (+)	
-Cheng & Furnham (2013) [36]	Correlation between an exercise score (1–6) and parental occupation (RGSC 1951: I, II, IIINM, IIIM, IV, V) with higher scores for higher occupational classes.	*r* = −0.020 (ns)		None
-UK; (NCDS)				
-5921; 50 year.				
-Juneau (2014) [37]	Correlation between LTPA (0–224 with 23 unique values) and parental occupation (RGSC: I, II, IIIN, IIIM, IV/V) with higher scores for lower occupational classes.	Age 0		None
-UK; 1970 British Cohort Study		♂: *r* = −0.080 (+, *p* < 0.001)		
-9624; 34 years.		♀: *r* = −0.053 (+, *p* < 0.001)		
		Age 5		
		♂: *r* = −0.048 (+, *p* < 0.001)		
		♀: *r* = −0.077 (+, *p* < 0.001)		
		Age 10		
		♂: *r* = −0.086 (+, *p* < 0.001)		
		♀: *r* = −0.064 (+, *p* < 0.001)		
	Parameter estimates from structural equation model (zero-inflated Poisson models) for LTPA by parental occupation at birth and ages 5 and 10.		Parental occupation at birth:	Occupational physical activity, transport-related physical activity
			Logistic portion of model:	
			♂ = 0.054 (ns)	
	(Results presented from an accumulation of risk with additive effects model (best fit), for results for ages 5 and 10 see paper.		♀ = 0.88 (*p* < 0.05)	
			Counts portion of model:	
			♂ = −0.049 (*p* < 0.05)	
			♀ = 0.050 (*p* < 0.05)	
-Osler (2008) [38]	Odds of sedentary leisure activity in low compared to high parental occupational class.		OR = 1.10 {0.97; 1.26}	Age
-Denmark; 1953 Metropolit Birth Cohort			OR = 0.90 {0.78; 1.05}	Age, own education, own occupational class, divorce, cognition
-6292^♂^; 51 year.				
-Jørgensen (2013) [41]	Prevalence of low LTPA in five parental occupational groups (1. higher professional 2. lower professional/non-routine M 3. self-employed 4. skilled blue-collar 5. unskilled blue-collar)	1–5:		None
-Denmark; Danish Health Care Worker Cohort		♀ = −5.7 % (+, *p* = 0.011)		
-1661^♀^; 35.4 years (mean)				
-Barnekow-Bergkvist (1998) [42]	Regression coefficients for LTPA MET hours/week. comparing non-manual to manual parental occupations.		β:	Own education, sport club member, two-hand lift, attitudes to soccer & handball
-Sweden			♂ = reported as ns	
			♀ = 0.18 (+)	
-278; 34 years.				
-Tammelin (2003) [43]	Odds of physical inactivity in parental occupational groups (1. skilled professional 2. skilled worker 3. unskilled worker 4. farmer) with skilled professional used as reference category.		Odds Ratios (4 vs. 1):	After-school sports
-Finland; 1966 North Finland Birth Cohort			♂ = 1.18 {0.94; 1.49} (ns)	
-7794; 31 year.			♀ = 0.80 {0.63; 1.02} (ns)	
-Makinen (2009) [44]	Odds of inactivity and moderate LTPA relative to high LTPA in father’s occupational groups (office employee, manual worker, self-employed, farmer) with office employee used a reference category.		ORs (farmer vs. office employee):	Age
-Finland; Health 2000 Survey			Inactivity (♂) = 1.69 (+)	
-6262; 30+ Yrs.			Inactivity (♀) = 0.97 (ns)	
			Moderate LTPA (♂) = 1.68 (ns)	
			Moderate LTPA (♀) = 1.08 (ns)	
-3905; 30+ Yrs.	Odds of inactivity and moderate LTPA relative to high LTPA in mother’s occupational groups (office employee, manual worker, self-employed, farmer) with office employee used a reference category.		ORs (farmer vs. office employee):	Age
			Inactivity (♂) = 1.49 (ns)	
			Inactivity (♀) = 0.87 (ns)	
			Moderate LTPA (♂) = 1.99 (ns)	
			Moderate LTPA (♀) = 1.40 (+)	
-Wichstrøm (2013) [45]	LTPA in five parental occupational groups (leader, high professional, low professional, manual, farmer/fisherman).	Reported as ‘unrelated to LTPA at any time point’ (ns)		None
-Norway				
-> 2800; 25–32 years				
-Peck (1994) [48]	Risk of no regular physical activity compared to the sample average in seven parental occupational groups (self-employed with employees, self-employed w/o employees, higher non-manual, assistant non-manual, skilled manual, unskilled manual, farmers).		Unskilled manual:	None
-Sweden			♂ = 1.24 (ns)	
-13,695; 16–74 years.			♀ = 1.24 (ns)	
			Higher non-manual:	
			♂ = 0.73 (ns)	
			♀ = 0.73 (ns)	
-Beunen (2004) [50]	Correlation and regression coefficients for sport, leisure-time and counts indices by parental occupation. Only leisure-time presented in paper.	Leisure-time:	Leisure-time:	Skeletal maturity, sum of skinfolds
-Belgium; Leuven Longitudinal Study of Flemish Boys		*r* = 0.13 (ns)	β at 16 years = 0.17 (+)	
-166^♂^; 40 year.			β at 18 years = 0.16 (+)	
-Kamphuis (2013) [52]	Prevalence of inactive, little and moderately active in three parental occupational groups (1. professional 2. white collar 3. blue collar).	1–3:		None
-Netherlands; GLOBE Study		Inactive = 1.5 % (ns)		
-4894^♂^; 40–75 years.		Little active = −0.9 % (ns)		
		Moderately active = 2 % (ns)		
-van de Mheen (1998) [53]	Odds of no LTPA and frequent LTPA by parental occupation (1. higher grade professional 2. lower grade professional/routine NM 3. self-employed 4. high/low skilled M 5. unskilled M) with higher grade professional used a reference category.		Odds Ratios (5 vs. 1):	Age, sex, religion, marriage, urbanisation
-Netherlands; Longitudinal Study on Socio-Economic Health Differences			No LTPA = 1.82 (+)	
			Frequent LTPA = 0.59 (+)	
-13,854; 25–74 years.				
			No LTPA = 1.62 (ns)	As above plus own occupational class
			Frequent LTPA = 0.68 (+ in ♀ only)	
-Regidor (2004) [49]	Prevalence and odds of physical inactivity in four parental occupational groups (1. professional, manager, proprietor, clerical worker 2. self-employed farmer 3. skilled/unskilled manual worker 4. paid farm worker) with professional group used as reference category.	1–4 (♂) = −9.5 % (+, *p* = 0.043)		None
-Spain		1–4 (♀) = −7.9 % (+, *p* = 0.011)		
-3658; 60+ Yrs.				
			Prevalence Ratios (4 vs. 1):	Age
			♂ = 1.29 {1.07; 1.56} (+, ns: 3 vs. 1)	
			♀ = 1.17 {1.03; 1.32} (+, ns: 2 vs. 1)	
			♂ = 1.28 (1.05; 1.55) (+, ns: 3 vs. 1)	Age, own occupational class
			♀ = 1.15 (1.01; 1.31) (+, ns: 2vs. 1)	
	Odds of physical inactivity in manual compared to non-manual parental occupations.		Manual vs. Non-manual:	Age
			♂ = 1.04 (0.91; 1.18) (ns)	
			♀ = 1.14 (1.05; 1.24) (+)	
			♂ = 1.03 {0.90; 1.17} (ns)	Age, own occupational class
			♀ = 1.12 {1.03; 1.23} (+)	
-Bowen (2010) [56]	Prevalence of vigorous exercise in manual (M) and non-manual (NM) parental occupations.	NM-M = 6 % (+, *p* < 0.001)		None
-US; Health & Retirement Study, Study of Asset & Health Dynamics, & two other cohorts				
-18,465; 51+ Yrs.				

^a^Both men and women included in analysis unless otherwise stated, *N* ^*♂*^ analytic sample consists of men only, *N* ^*♀*^ analytic sample consists of women only

^b^ *LTPA* leisure-time physical activity, *MET* metabolic equivalent, *RGSC* Registrar General’s Social Classification (I: professional, II: managerial and technical, IIIN: skilled non-manual, IIIM: skilled manual, IV: partly skilled, V: unskilled), *M* manual, *NM* non-manual

^c^For brevity, prevalence of LTPA shown as crude difference between named childhood SEP groups, along with measure of precision (95 % confidence intervals where available unless stated otherwise), *SE* standard errors, *r* correlation coefficient, *OR* odds ratio from logistic regression, *β*: regression coefficient, “+” Statistically significant (*p* ≤ 0.05) association between less advantaged childhood SEP and less frequent adult LTPA, “−” Statistically significant (*p* ≤ 0.05) association between less advantaged childhood SEP and more frequent adult LTPA, *ns* Statistically non-significant association (*p* > 0.05) between childhood SEP and adult LTPA

^d^ *BMI* body mass index, *CVD* cardiovascular disease, *CHD* coronary heart disease

**Table 3:** Results of studies testing the association between parental education and leisure-time physical activity (LTPA) in adults

-1st author (year)	How results presented and interpretation^b^	Correlation coefficient/ difference in prevalence^c^	Estimates from statistical modelling^c^	Adjustments^d^
-Country; study name				
-Sample size^a^; age				
-Johnson (2011) [20]	Correlation and regression coefficients for 6-point LTPA score and years of parental education.	*r* = 0.08 (+)		None
-UK; Lothian Birth Cohort 1936			β = 0.03 (ns)	Own education, own occupational class & more
-1091; 70+ Yrs.				
-Silverwood (2012) [33]	Prevalence of LTPA (low/gardening/sport & leisure), walking and cycling during work & for pleasure (high, low) in four groups of paternal education (1. secondary and greater 2. secondary only or primary and further education or greater 3. primary and further education with no qualifications attained 4. primary only).	1–4:		None
-UK; MRC National Survey of Health & Development		Sport & leisure (♂) = 14.5 %		
		(+, *p* < 0.001)		
-≥ 3100; 31–53 years.		Sport & leisure (♀) = 20.9 %		
		(+, *p* < 0.001)		
		Walking (High) (♂) = −21.6 %		
		(−, *p* < 0.001)		
		Walking (High) (♀) = −8.8 %		
		(−, *p* < 0.001)		
-Kuh & Cooper (1992) [34]	Prevalence of most active in sports & recreational activities in 4 groups of parental education (1. secondary & greater 2. secondary only or primary & further education or greater 3. primary & further education with no qualifications attained 4. primary only).	1–4:		None
-UK; MRC NSHD		♂ (father) = 12 % (+, *p* < 0.01)		
-> 2850; 36 years.		♀ (father) = 21.3 % (+, *p* < 0.001)		
		♂ (mother) = 2 % (+, *p* < 0.001)		
		♀ (mother) = 19 % (+, *p* < 0.001)		
-2144; 36 years.	Odds of most active in sport & recreational activities comparing three highest groups of maternal education to the lowest group.		Odds Ratios:	
			1 vs. 4 = 1.24 (0.99; 1.55} (ns)	Own education, sex, childhood health, personality, and ability at games
			2 vs. 4 = 1.52 (1.22; 1.91} (+)	
			3 vs. 4 = 1.24 (1.02; 1.50} (+)	
-Pinto Pereira (2014) [35]	Odds of low LTPA comparing those with two minimally schooled parents to those without.		Odds Ratios:	
-UK; National Child Development Study 1958 (NCDS)			age 33 = 1.26 {1.15; 1.37} (+)	None
			Age 42 = 1.28 {1.18; 1.38} (+)	
-12,776 had ≥ one measure of LTPA; 33, 42, 50 year.			Age 50 = 1.42 {1.29; 1.57} (+)	
			Age 33 = 1.14 {1.04; 1.26} (+)	Sex
			Age 42 = 1.13 {1.03; 1.24} (+)	
			Age 50 = 1.22 {1.10; 1.35} (+)	
			Age 33 = 1.05 {0.95; 1.16} (ns)	Sex, parental education, aptitude household amenities, cognition, lifestyle factors age 16, & more
			Age 42 = 1.03 {0.94; 1.13} (ns)	
			Age 50 = 1.13 {1.01; 1.25} (+)	
			Age 33 = 1.02 {0.92; 1.13} (ns)	As above plus own education, own social class, BMI, mental health, number of children in the household, limiting illness
			Age 42 = 1.00 {0.91; 1.10} (ns)	
			Age 50 = 1.07 {0.96; 1.19} (ns)	
-Kvaavik (2011) [40]	Regression coefficients for LTPA per increase in parental education (college/university/>12 years, high/comprehensive school/12 years, high school/10 year, 1 year of technical college/8–9 years, elementary school/7 years).		β (estimated from figures):	
-Norway; Oslo Youth Study			Age 25 (father) ≈ 0.06 (ns)	Sex, whether participated in school health education intervention
-240–407^♂^; 25, 33, 40 year.			Age 33 (father) ≈ 0.12 (+)	
			Age 40 (father) ≈ 0.01 (ns)	
			Age 25 (mother) ≈ 0.05 (ns)	
			Age 33 (mother) ≈ 0.12 (+)	
			Age 40 (mother) ≈ −0.06 (ns)	
			Age 25 (father) ≈ 0.01 (ns)	As above plus own education
			Age 33 (father) ≈ 0.05 (ns)	
			Age 40 (father) ≈ 0.01 (ns)	
			Age 25 (mother) ≈ −0.01 (ns)	
			Age 33 (mother) ≈ 0.06 (ns)	
			Age 40 (mother) ≈ −0.01 (ns)	
-Makinen (2009) [44]	Odds of inactivity and moderate LTPA relative to high LTPA by parental education (secondary, middle, primary) with secondary education used as reference category.		ORs (primary vs. secondary):	Age
-Finland; Health 2000 Survey			Inactivity (♂) = 1.10 (ns)	
-6492; 30+ Yrs.			Inactivity (♀) = 1.56 (+)	
			Moderate LTPA (♂) = 1.45 (ns)	
			Moderate LTPA (♀) = 1.37 (ns)	
-Leino (1999) [46]	Prevalence of physical inactivity in three groups of parental education (1. >12 years 2. 9–12 years 3. <9 years).	1–3 (♂) = −14.7 % (ns)		Age
-Finland; Cardiovascular Risk in Young Finns Study		1–3 (♀) = −9.2 % (ns)		
-432; 21–30 year.				
-Osler (2001) [47]	Odds of low LTPA comparing the two highest groups of parental education to the lowest group (1. ≥ 9 years 2. 8–9 years 3. <7 years).		Odds Ratios (1 vs. 3):	None
-Denmark; offspring of Copenhagen City Heart Study (CCHS).			♂ = 1.3 {0.6; 3.0} (ns)	
			♀ = 0.5 {0.2; 1.1} (ns)	
-317; 19–31 year.				
			♂ = 0.7 {0.4; 3.2} (ns)	Age, own education, own occupational class, smoking status
			♀ = 0.6 {0.2; 2.4} (ns)	
-Beunen (2004) [50]	Correlation between sports, leisure-time and counts indices of physical activity and parental education.	r (sport, father) = 0.17 (+)		None
-Belgium; LLSFB		r (sport, mother) = 0.14 (ns)		
-166^♂^; 40 year.		r (leisure-time, father) = 0.14 (ns)		
		r (counts, mother) = 0.15 (ns)		
	Regression coefficients for sport, leisure-time and counts indices of physical activity per increase in years of parental education		β (sport, father) = 0.19 (+)	Stature (sport index)
			β (leisure-time, father) = 0.14 (+)	Stature, pulse recovery (leisure-time index)
-Wray (2005) [55]	Odds of low physical activity per unit increase (0–17) in years of parental education.		Odds Ratios:	
-US; Health & Retirement Study (HRS); Study of Asset & Health Dynamics (AHEAD)			HRS = 0.964 (+, *p* ≤ 0.001)	Age, gender, ethnicity, marriage, interactions
			AHEAD = 0.878 (+, *p* ≤ 0.001)	
-6106; 51–61 year (HRS), 3636; 70+ Yrs. (AHEAD)			HRS = 0.976 (+, *p* ≤ 0.05)	As above plus own education, economic resources
			AHEAD = 0.910 (+, *p* ≤ 0.05)	
-Bowen (2010) [56]	Prevalence of vigorous exercise in two groups of parental education (1. > 8 years 2. ≤ 8 years).	1–2 (father) = 4 % (+, *p* ≤ 0.001)		None
-US; HRS,AHEAD & more		1–2 (mother) = 4 % (+, *p* ≤ 0.001)		
-18,465; 51+ Yrs.				
-Phillips (2009) [61]	Correlation between exercise kilocalories/week. and years (1–24) of parental education.	*r* = 0.084 (+)		None
-US; Health & Behaviour Project				
-811; 30–54 years.				
-Frank (2003) [58]	Prevalence of exercise in six groups of parental education (1. medical school 2. graduate school 3. college graduate 4. some college 5. high school 6. < High school) and three groups of both parent’s education) (1. Both ≥ graduate school 2. mix 3. Both ≤ graduate school).	1–6 (father) = 2 % (ns)		None
-US; Women Physician Health Study		1–6 (mother) = −4 % (ns)		
-2884^♀^; 30–70 year.		1–3 (both) = 5 % (ns)		
-Gall (2010) [64]	Prevalence of LTPA by level of parental education (1. high 2. medium 3. low).	1–3:		None
-Australia; Childhood Determinants of Adult Health Study		♂ = 3 % (ns)		
		♀ = 1 % (ns)		
-1973; 26–36 years.				

^a^Both men and women included in analysis unless otherwise stated, *N* ^*♂*^ analytic sample consists of men only, *N* ^*♀*^ analytic sample consists of women only

^b^ *LTPA* leisure-time physical activity

^c^For brevity, prevalence of LTPA shown as crude difference between named childhood SEP groups, along with measure of precision (95 % confidence intervals where available unless stated otherwise), *SE* standard errors, *r* correlation coefficient, *OR* odds ratio from logistic regression, *β* regression coefficient, “+” Statistically significant (*p* ≤ 0.05) association between less advantaged childhood SEP and less frequent adult LTPA, “−” Statistically significant (*p* ≤ 0.05) association between less advantaged childhood SEP and more frequent adult LTPA, *ns* Statistically non-significant association (*p* > 0.05) between childhood SEP and adult LTPA

^d^ *BMI* body mass index

**Table 4:** Results of studies testing the association between indices and other measures of childhood socioeconomic position and leisure-time physical activity (LTPA) in adults

-1st author (year)	How results presented and interpretation^b^	Correlations coefficient/difference in prevalence^c^	Estimates from statistical modelling^c^	Adjustments^d^
-Country; study name				
-Sample size^a^; age				
-Johnson (2011) [20]	Correlation and regression coefficients for a 6-point LTPA score and an index of childhood household amenities.	*r* = 0.00 (ns)		None
-UK; Lothian Birth Cohort 1936			β = 0.02 (ns)	Own education, own occupational class & more
-1091; 70 year.				
-Hilsdon (2008) [22]	Prevalence of four indicators of childhood household amenities and car access in 4 groups of frequency of physical activity hours/week.	≥3–0 (hours/week.):		None
-UK; British Women’s Heart & Health Study (BWHHS)		Shared bedroom = −7.7 % {−5.9; −8.7} (+)		
		No indoor toilet = −8.8 % {−7.9;- 9.8} (+)		
-> 4100^♀^; 60–79 years.		No hot water = −9.6 % {−8.6; −10.4} (+)		
		No car access = −7.9 % {−6.8; −9.1} (+)		
	Odds of more frequent physical activity per unit increase in childhood SEP (parental occupation, household amenities and car access) with higher scores representing more adversity.		OR = 0.85 {0.81; 0.89} (+)	Age
			OR = 0.93 {0.89; 0.98} (+)	Age, adult SEP, area deprivation.
			OR = 0.94 {0.90; 0.99} (+)	As above plus smoking, BMI, CVD, respiratory disease
-Watt (2009) [23]	Difference in prevalence of low exercise between those reporting no and those reporting yes to questions on childhood household amenities and car access.	Shared bedroom = 5.4 % {1.9; 9.0} (+)		None
-UK; BWHHS		No hot water = 6.1 % {2.4; 9.8} (+)		
-3523^♀^; 60–79 years.		No indoor toilet = 6.8 % {3.1; 10.4} (+)		
		No car access = 7.9 % {3.3; 12.4} (+)		
	Odds of low exercise per unit increase in childhood SEP with higher scores representing more adversity.		OR = 1.12 {1.07; 1.17} (+)	None
			OR = 1.06 {1.01; 1.12} (+)	Age, own adult SEP
-Pinto Pereira (2014) [35]	Odds of low LTPA per unit increase (0–18) on index of childhood household amenities (access to bathroom, indoor lavatory and hot water, with higher scores indicating more limited access).		Odds ratios:	None
-UK; National Child Development Study 1958 (NCDS)			Age 33 = 1.03 {1.01; 1.04} (+)	
			Age 42 = 1.03 {1.01; 1.04} (+)	
-12,776 had ≥ one measure of LTPA; 33, 42, 50 year.				
			Age 50 = 1.04 {1.03; 1.05} (+)	
			Age 33 = 1.02 {1.001; 1.03} (+)	Sex
			Age 42 = 1.01 {0.999; 1.03} (ns)	
			Age 50 = 1.02 {1.01; 1.04} (+)	
			Age 33 = 1.01 {0.995; 1.03} (ns)	Sex, parental education, household amenities, cognition, aptitude, lifestyle factors at age 16, & more
			Age 42 = 1.01 {0.99; 1.02} (ns)	
			Age 50 = 1.02 {1.002; 1.03} (+)	
			Age 33 = 1.01 {0.99; 1.02} (ns)	As above plus own education, own social class, BMI, mental health, number of children in the household, limiting illness
			Age 42 = 1.01 {0.99; 1.02} (ns)	
			Age 50 = 1.01 {0.999; 1.03} (ns)	
-Lynch (1997) [39]	Prevalence of conditioning inactivity & low quartile of conditioning activity by an index of parental occupation, parental education & more (1. high 2. middle 3. poor).	No conditioning activity:		Age
-Finland; Kuopio Ischaemic Heart Disease Risk Factor Study		1–3 = −0.4 % (ns)		
		Low quartile:		
-2682^♂^; 42–60 year.		1–3^+^ = −5.7 % (+)		
-Makinen (2009) [44]	Odds of inactivity and moderate LTPA relative to high LTPA for those reporting yes to long-term financial problems; regular parental unemployment.		Odds Ratios (inactivity):	Age
-Finland; Health 2000 Survey			♂ = 1.04 (ns); 1.35 (ns)	
-6492; 30+ Yrs.			♀ = 1.18 (ns); 1.45 (ns)	
			Odds Ratios (moderate LTPA):	
			♂ = 0.95 (ns); 1.31 (ns)	
			♀ = 1.13 (ns); 1.36 (ns)	
-Beunen (2004) [50]	Correlation and regression coefficients for sport, leisure-time and counts indices per increase in urbanisation score of the childhood home. Only counts results presented in paper.	Counts:	Counts:	
-Belgium; LLSFB		*r* = 0.18 (+)	β at 14 years = 0.17 (+)	Sit reach, pulse recovery, sports participation (regression)
-166^♂^; 40 year.			β at 16 years = 0.15 (+)	
			β at 18 years = 0.15 (+)	
-Scheerder (2006) [51]	Path coefficients for level of sports participation based on an index of parental occupation and parental education (lower class, middle class, upper class).		β from path model = −0.07 {−0.22; 0.08} (ns)	Age, own education, own occupational class, BMI, parent’s sport, & more
-Belgium; Leuven Longitudinal Study of Flemish Girls (LLSFG)				
-234^♀^; 32–41 year.				
-Pudrovska (2013) [54]	Path coefficients for exercise per change in index of parental occupation, parental education, family income, father’s occupational income and occupational education.		‘Total effects’	None
-US; 1957 Wisconsin Longitudinal Study			β = 1.117 (+, *p* < 0.001)	
-5778; 65 years.				
			‘Direct effects’	Marriage, children, alcohol use, smoking status, own SES, health, obesity, depression
			♂ = 0.211 (+, *p* < 0.01)	
			♀ = 0.091 (+, *p* < 0.05)	
			♂ = 0.018 (ns)	As above plus high school sports
			♀ = 0.039 (ns)	
-Carroll (2011) [57]	Correlation between physical activity kilocalories/week. and a 6-point index of household amenities and car access (for every 2 years, up to age 18).	r (range) = −0.15 to 0.14 (ns)		None
-US; Vaccination Immunity Project				
-112; 40–60 year.				
-Tsenkova (2014) [59]	Regression coefficients for more frequent vigorous exercise per increasing disadvantage on a 6-point index of parental education, childhood welfare status and financial circumstances.		β = −0.11 {SE = 0.03} (+, *p* < 0.01)	Age, sex, race, smoking history.
-US; Midlife in the US Study.				
-895; 25–74			β = −0.08 {SE = 0.03} (+, *p* < 0.05)	As above plus adult SEP
-Kern (2010) [60]	Regression coefficients for physical activity per unit increase in standardised index of parental occupation and education.		β (Physical activity level):	None
-US; Terman Life Cycle Study			♂ = −0.03 {SE = 0.02} (ns)	
-1114;25–61 year.			♀ = 0.02 {SE = 0.01} (ns)	
-Schooling (2007) [62]	Prevalence of HEPA^b^, minimally active, and inactive in three groups of (3 items, 1 or 2 items, 0 parental possessions during childhood	HEPA-inactive:		None
-China; Guangzhou Bio-bank Cohort Study (GBCS)		♂ (0 items) = 6.1 % (−, *p* < 0.01)		
		♀ (0 items) = −3.2 % (*p* < 0.01)		
-9748; 50+ Yrs.				
-Elwell-Sutton (2011) [63]	Prevalence of HEPA^b^, minimally active, and inactive in those reporting 1–3 items or 0 parental possessions during childhood).	HEPA-inactive:		None
-China; GBCS		0 Items = −0.17 % (ns)		
-20,086; 50+ Yrs.		1–3 items = 0.61 % (ns)		

^a^Both men and women included in analysis unless otherwise stated, *N* ^*♂*^ analytic sample consists of men only, *N* ^*♀*^ analytic sample consists of women only

^b^ *LTPA* leisure-time physical activity, *HEPA* Health enhancing physical activity–acronym used in the two GBCS papers [62, 63]

^c^For brevity, prevalence of LTPA shown as crude difference between named childhood SEP groups, along with measure of precision (95 % confidence intervals where available unless stated otherwise), *SE* standard errors, *r* correlation coefficient, *OR* odds ratio from logistic regression, *β* regression coefficient; “+” Statistically significant (*p* ≤ 0.05) association between less advantaged childhood SEP and less frequent adult LTPA, “−” Statistically significant (*p* ≤ 0.05) association between less advantaged childhood SEP and more frequent adult LTPA, *ns* Statistically non-significant association (*p* > 0.05) between childhood SEP and adult LTPA

^d^ *BMI* body mass index, *CVD* cardiovascular disease

### Parental occupational class

Thirty papers (22 studies) tested the association between parental occupation during childhood and adult LTPA and twenty-one (16 studies) found evidence that a lower parental occupational class was associated with less frequent LTPA during adulthood (Table 2). All UK studies used the Registrar General’s Social Classification (RGSC) to categorise parental occupations into usually four or two groups. Studies from other countries used similar categorisations to those of the RGSC although several considered farming occupations as separate groups [43–45, 48, 49].

Evidence was available from three British birth cohorts initiated in 1946, 1958 and 1970. A higher prevalence of sports participation in higher paternal occupational groups was reported at age 36 in women from the MRC National Survey of Health and Development (NSHD) [34]. Later findings from this cohort [33] showed similar trends for LTPA derived by latent classes in both men and women and trends in the opposite direction for a combined walking during work and pleasure outcome (Table 2). Gender-adjusted analyses from the next oldest cohort born in 1958, the National Child Development Study (NCDS), showed that a lower parental occupational class was associated with less LTPA at ages 33, 42 and 50 years [35]. This association was fully attenuated at age 33 after accounting for other early life factors and following further adjustments (including for own adult SEP), it was only seen at age 50 [35]. A second NCDS paper reported a non-significant correlation between parental occupation and exercise at age 50 [36]. Father’s occupational class measured three times during early life was associated with LTPA at age 34 in the 1970 British cohort study [37].

Manual father’s occupation was associated with less LTPA and more inactivity in men [24, 25] and women [21–23] from the British Regional Heart Study (BRHS) and British Women’s Heart and Health Study (BWHHS) respectively. After accounting for age and own occupational class [21, 25], this association was only found in the BWHHS [21]. A higher prevalence [28] and higher odds [26] of inactivity were reported in lower parental occupational groups of the Whitehall II Study. This association was considerably attenuated and no longer significant following adjustment for adult SEP [28] and a third paper from this cohort reported no difference in levels of inactivity between manual and non-manual parental occupations [27]. Findings from the West of Scotland Collaborative Study suggest less exercise in lower parental occupations [29, 30]. A weak correlation between higher paternal occupational groups and more LTPA was reported in the Lothian Birth cohort 1936 (LBC1936) and no association was found in analysis adjusted for adult SEP [20]. In the Mid-span family study, manual and non-manual groups did not differ by levels of inactivity [32], but the prevalence of sports and exercise was higher in higher father’s occupational groups of a Scottish survey [31].

Several Scandinavian studies reported null findings including Danish [38] and Finnish [43] birth cohorts, a Norwegian study [45], and an analysis of 34 year old Swedes [42]. The latter [42] found that women but not men from non-manual paternal backgrounds spent more metabolic equivalent hours/week in LTPA compared with those of manual father’s occupations. Higher and lower father’s occupational groups were less and more respectively, inactive than the mean level of activity of employed Swedes [48], but this was not tested at a high significance level (*p* < 0.10) [48]. Mostly null findings were reported in the Finnish Health 2000 Survey [44] however, men from lower paternal occupational groups were found to be more inactive than those from higher groups and women with mothers in manual occupations were more likely to be only moderately active compared with daughters of office employee mothers [44].

Dutch adults living near Eindhoven from lower paternal occupational strata were more likely to be inactive and less likely to be frequently active during leisure-time compared with those from professional backgrounds [53]. After accounting for own occupational class, this association remained for frequent LTPA and in women only [53]. A Dutch study that only included men from Eindhoven [52] found no difference by parental occupation in the prevalence of activity [52]. Belgian men’s paternal occupational class was associated with their leisure-time but not sports or accelerometer indices [50]. Age-adjusted findings from an older Spanish sample showed that lower father’s occupational groups were more likely to be inactive than higher groups and the association was more evident in women following adjustment for own occupational class [49]. Compared with the manual group, non-manual father’s occupational groups of a large US sample had a higher prevalence of vigorous exercise [56].

### Parental education

Fourteen papers (13 studies) presented associations for parental years or level of education and ten (9 studies) found evidence of an association between lower levels of parental education and less frequent LTPA in adults (Table 3).

Similar trends to those found for occupation were reported in the NSHD, i.e. less LTPA [33, 34] and more walking (during work and pleasure) [33] in lower parental educational groups. Analysis adjusted for own education showed that those with more highly educated mothers were more active in sports at age 36 [34] but no difference was found when the highest maternal educational group was compared to the lowest (Table 3). Gender-adjusted NCDS analyses comparing those without and with two minimally educated parents showed that the latter were more likely to be physically inactive at ages 33, 42 and 50 years [35]. This association was fully attenuated at ages 33 and 42 after other early life factors were included in the analysis and likewise at age 50 following further adjustments including for own adult SEP (Table 3). More parental years in education were weakly correlated with more LTPA in LBC1936 but adjusted analysis did not find an association [20].

Parental education was unrelated to leisure-time inactivity in a Danish sample [47], and null-findings were reported by two Finnish studies [44, 46]. However, one of the latter [44] found that Finnish women with a primary-level educated parent were more likely to be inactive compared with those with a secondary-level educated parent [44]. LTPA (at age 33 only) was linked to parental education in a Norwegian study but not after adjustment for own education [40]. Belgian men’s father’s education was related to their self-reported sports and leisure-time activity but not accelerometer indices [50]. A lower parental education in US adults was associated with less prevalent vigorous exercise [56] and with higher adjusted-odds of low exercise [55]. Higher parental education was correlated with higher estimated exercise energy expenditure in a Pennsylvanian sample [61]. Three measures of parental education were unrelated to exercise in women physicians born in the US [58] and there was no association between parental education and LTPA in an Australian study [64].

### Indices and other measures of childhood SEP

Fourteen papers (12 studies) tested associations between indices and other measures of childhood SEP and adult LTPA and seven (6 studies) found an association between a lower childhood SEP and less frequent LTPA in adulthood (Table 4).

An index measuring household characteristics and car access during childhood was unrelated to LTPA in LBC1936 [20] but four measures of housing characteristics and car access were each associated with LTPA in the BWHHS [22, 23]. Combining these four indicators and paternal occupation into a summary variable showed that with increasing childhood socioeconomic adversity, women were more likely to be low exercisers [23] and less likely to be more physically active [22]. Having more limited household amenities was related to leisure-time physical inactivity at ages 33, 42 and 50 years in NCDS, but not at age 42 when gender was taken into account [35]. After adjustment for a range of early life factors, this association was only found at age 50 and associations were considerably attenuated and were no longer observed at any age following the addition of adult covariates, including own SEP, to the analysis (Table 4).

Compared with those ranked middle or poor on an index of parental occupation, education, external perceptions of wealth, and housing characteristics, Finnish men who ranked high on the index were less likely to be in the lowest quartile of conditioning activities [39]. No difference was found when the prevalence of inactivity was compared in this sample [39]. A different Finnish study reports no association between long-term financial problems or regular parental unemployment and LTPA in adults [44]. More urban locations of Belgian men’s childhood homes were related to higher accelerometer counts but not to any self-reported outcomes [50]. An index of parental occupation and education was not associated with Belgian women’s sports participation [51].

Increasing disadvantage as indicated by an index of parental education, childhood welfare status and financial level growing up was associated with less participation in vigorous exercise in US adults [59]. This association was only partially attenuated following adjustment for own adult SEP [59]. An index of parental occupation and education was unrelated to activities and hobbies of a Californian sample [60], but in older US adults [54] a higher childhood SEP, indicated by a similar index that included parental income, was associated with more exercise at age 65. The authors tested the role of mediating factors and report that own SEP explained almost half of this association [54]. No correlation was found in 112 US participants between a similar index and estimated activity energy expenditure [57]. Findings from the GBCS suggest a higher prevalence of inactivity (and a lower prevalence of LTPA) in Chinese participants reporting more parental possessions during their childhood [62, 63].

## Discussion

### Summary of results

This systematic review included 45 papers from 36 study samples and found evidence of less frequent LTPA in adults from less advantaged childhood socioeconomic backgrounds. Twenty-two studies report results that associate a lower childhood SEP with less frequent adult LTPA; thirteen studies report no association. 9/16 studies that presented results adjusted for own adult SEP reported statistically significant associations between childhood SEP and adult LTPA [21–23, 34, 35, 42, 49, 53–55, 59]. Studies presenting results before and after adjustment for adult SEP found that accounting for own SEP in adulthood typically partly attenuated associations (Tables 2, 3, 4). Gender-stratified analyses showed more evidence of an association in women compared with men [34, 42, 44, 49, 53]. 1/10 UK [32], 3/8 US [57, 58, 60] and 6/11 Scandinavian [38, 43, 45–48] studies found no evidence of childhood socioeconomic differences in adults’ LTPA. Findings did not differ systematically by type of childhood SEP indicator or age at assessment of LTPA.

### Explanation of findings

Existing reviews link a lower childhood SEP to a range of disadvantageous adult outcomes, including physical capability [65], cardiovascular disease [66] and mortality [67]. Reviews focusing on different life stages have shown that from childhood through to old age, in cross-sectional analyses, lower socioeconomic groups tend to participate less in LTPA than more advantaged groups [7–9]. In addition to participating less in LTPA during childhood [7], a study of over 2000 Dutch adults provides evidence that children from lower socioeconomic backgrounds have a lower likelihood of initiating a sport throughout their lives [68].

One possible reason for finding an association between a lower childhood SEP and less frequent adult LTPA is due to the continuity of SEP across life. A lower childhood SEP tends to restrict future SEP [69], partly by predisposing to social pathways operating across life which can limit educational opportunities and ultimately socioeconomic potential, e.g. in occupational class, income and wealth [10]. These pathways can influence the availability of, and a person’s response to, opportunities for the development of LTPA [10].

**Fig. 2:**
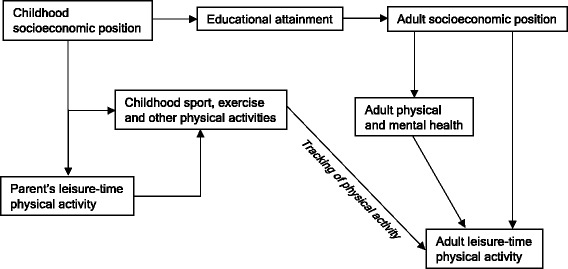
Hypothesised pathways explaining associations found between childhood socioeconomic position and adult leisure-time physical activity

Associations between childhood SEP and adult LTPA were reported in several analyses which were adjusted for own adult SEP [21–23, 34, 35, 42, 49, 53–55, 59] suggesting that complementary pathways are likely to be involved (Fig. 2). Participation in sports and exercise in early life tends to be socioeconomically patterned [7] and tracks into adulthood [70], potentially forming an important determinant of adult LTPA. Since adult LTPA also displays a socioeconomic gradient [8, 9], less socioeconomically advantaged children are likely to have less physically active parents who may in turn unfavourably influence their own children’s involvement in LTPA [71]. Childhood socioeconomic circumstances may influence the acquisition of sets of interpersonal skills such as decision making, self-efficacy and self-esteem which can help people maintain health behaviour such as LTPA [72]. Socioeconomic differences in children’s growth and motor development [73] could contribute to differences in subsequent LTPA.

Thirteen studies presenting only null findings do not support the review’s hypothesis [32, 38, 43, 45–48, 51, 52, 57, 58, 60, 64]. Participation in sports and exercise is linked to a range of factors other than SEP, including genetics [74], life transitions, culture and policy [75], some of which could play a greater role in determining participation. Evidence for less tracking of LTPA when compared with other health behaviours such as sedentary behaviour [76] supports this argument although measurement error could explain the lower tracking of LTPA [70]. Associations may vary by setting and cohort due to varying influences on LTPA by these factors and are also likely to be influenced by study quality.

### Sources of heterogeneity

Inconsistent findings could be due to differences between studies including in design and risk of bias. Despite an overall medium study quality, considerable variation between studies in the assessment and formulation of LTPA (Table 1) and adjustment for potential confounders (Tables 2, 3, 4) can influence associations. Small sample sizes [47, 50, 51] may lead to underpowered studies while multiple tests [40, 44, 50] risk detecting false associations. Lack of an association in men from the BRHS after accounting for adult SEP [25], and other reported null findings [30, 32], may be due to heterogeneity within childhood SEP groups as a result of using dichotomous indicators. Null findings from the Women Physician Health Study [58] might reflect insufficient variation in childhood socioeconomic background.

We did not find that results varied by the method of ascertainment of childhood SEP however, using recalled measures of childhood SEP can underestimate associations [77]. There was little evidence that the type of childhood SEP indicator used was a source of heterogeneity, suggesting that each indicator sufficiently captures the same underlying construct or that the various aspects of SEP are equally important. This is a similar observation to that of a previous review of European adults [9] but contrary to an earlier and geographically wider review which found education to be more strongly associated with contemporaneous LTPA [8].

Genuine gender differences in the association between childhood SEP and adult LTPA might exist. Like some studies in this review, a previous review found more evidence of an association in women than men between adult SEP and LTPA [8]. Absence of a gender difference in how childhood SEP relates to adult’s capacity to undertake exercise [65] suggests that the gender differences found in this review are likely explained by social rather than biological pathways, such as differences in risk factors which could impact on subsequent LTPA [78].

The tendency for Scandinavian studies to find less evidence of association compared with UK studies might be due to less variation between socioeconomic groups in Scandinavian cohorts than in the UK [79]. There could also be differences in the meaning of occupation between these settings, e.g. in Scandinavian cohorts, where there was more prevalent farming occupations [43–45, 48], SEP could be indicating how urban or rural is the environment, which may be independently related to LTPA [80].

More walking during work and pleasure in lower childhood SEP groups of the NSHD [33] might be explained by the inclusion of work-related PA as part of the outcome, which can be inversely associated with SEP [9]. Socioeconomic patterns of LTPA that are different to those usually observed in Western countries have been documented in China [81], which could explain the GBCS findings [62]. Other cohort, period and cultural differences might explain some of the between-study heterogeneity.

### Implications of findings

Due to heterogeneity in findings, a better understanding of how childhood SEP relates to adult LTPA is required. Future studies should use prospectively ascertained indicators of childhood SEP where this is feasible and examine validated and reliable measures of LTPA. Data from PA monitors could be used in conjunction with questionnaires to derive more holistic LTPA variables that capture parameters such as activity type, energy expenditure and time of day/week that activity is performed [82]. Strategies for maximising participant retention in long-running studies should be considered so as to minimise bias due to loss to follow-up. Within-individual levels of LTPA can vary over time and future research could in addition explore associations with patterns or change in LTPA, as well as different types of LTPA. To better characterise how associations vary by time and place, age, country, cohort and period differences should be formally tested while accounting for methodological differences. Testing hypothesised pathways (Fig. 2) can aid our knowledge of how childhood SEP relates to adult LTPA.

Despite the inconsistencies described, childhood socioeconomic circumstances can influence health throughout life and interventions to improve them will likely lead to additional benefits besides increased adult LTPA. As well as improving early life circumstances, intervening to promote adult LTPA could be one means to cut the link between a disadvantaged childhood SEP and poor adult health. Effectively promoting adult LTPA amongst those disadvantaged in childhood may in turn require a better understanding of the mechanisms linking childhood disadvantage to adult LTPA.

### Strengths and limitations of the review

Strengths of this review are the systematic process followed to locate and extract data from eligible studies and the searching of multiple databases and reference lists. Independently working researchers helped reduce the potential for errors in study screening, data extraction and quality assessment. Limitations include search restrictions to English language and to journal publications, which may introduce publication bias. The fact that presented results were not sufficiently comparable to be combined in a meta-analysis could be considered a limitation and this also meant we could not formally assess publication bias. However, the inclusion of all studies even where the review’s question was not the primary aim, and the findings of no association between childhood SEP and adult LTPA in 13 studies suggests publication bias is unlikely.

## Conclusions

This systematic review found evidence of an association between a less advantaged SEP in childhood and less frequent LTPA in adults (particularly among women and in UK cohorts) but considerable heterogeneity between studies was detected. Future studies should examine more detailed measures of LTPA, investigate underlying pathways and explore country differences. The findings suggest the need to provide additional opportunities and support to enable children from socioeconomically disadvantaged backgrounds to develop and maintain more active leisure pursuits and participate in sports and exercise across life.

## Additional files

Additional file 1:
**Search terms.**
Additional file 2:
**Data extraction form.**
Additional file 3:
**Quality assessment form.**

## Abbreviations

PA: Physical activity
LTPA: Leisure-time physical activity
SEP: Socioeconomic position
PRISMA: Preferred Reporting Items for Systematic Reviews and Meta-Analysis
