# Supplementary material for: Childhood socioeconomic position and adult leisure-time physical activity: a systematic review
Source: Int J Behav Nutr Phys Act. 2015 Jul 3;12:92. doi: 10.1186/s12966-015-0250-0 (PMC4501082; doi:10.1186/s12966-015-0250-0)
Supplement: Additional file 1: — Search terms. [file 12966_2015_250_MOESM1_ESM.pdf]

Additional file 1a OvidSP (Embase, Medline and PsycINFO) search strategy.

**Search Terms**
**1.** (physical* activ*)
**2.** (leisure adj3 time)
**3.** (sport*)
**4.** (exercise)
**5.** (walk*)
**6.** (recreational)
**7.** (father* adj3 (occupation* or education*))
**8.** (mother* adj3 (occupation* or education*))
**9.** (parent* adj3 (occupation* or education*))
**10.** (father* adj3 (income or manual))
**11.** (mother* adj3 (income or manual))
**12.** (parent* adj3 (income or manual))
**13.** (father* adj3 (social class or social status))
**14.** (mother* adj3 (social class or social status))
**15.** (parent* adj3 (social class or social status))
**16.** (child* adj3 (social class or social status))
**17.** (early-life adj3 (social class or social status))
**18.** (adolescen* adj3 (social class or social status))
**19.** (father* adj3 (socioeconomic or socio-economic))
**20.** (mother* adj3 (socioeconomic or socio-economic))
**21.** (parent* adj3 (socioeconomic or socio-economic))
**22.** (child* adj3 (socioeconomic or socio-economic))
**23.** (adolescen* adj3 (socioeconomic or socio-economic))
**24.** (early adj3 (socioeconomic or socio-economic))
**25.** (early-life adj3 (socioeconomic or socio-economic))
**26.** (child* adj3 (deprivation or poverty))
**27.** (early-life adj3 (deprivation or poverty))
**28.** (adolescen* adj3 (deprivation or poverty))
**29.** (child* adj3 overcrowding)
**30.** (adult*)
**31.** (midlife or mid-life)
**32.** (old*)
**33.** (later-life)
**34.** 1 OR 2 OR 3 OR 4 OR 5 OR 6
**35.** 7 OR 8 OR 9 OR 10 OR 11 OR 12 OR 13 OR 14 OR 15 OR 16 OR 17 OR 18 OR 19 OR 20 OR 21 OR 22 OR 23 OR 24 OR 25 OR 26 OR 27 OR 28 OR 29
**36.** 30 OR 31 OR 32 OR 33
**37.** 34 AND 35 AND 36
**38.** Limit 37 to humans
**39.** Remove duplicates from 38

Adj3 locates results where search terms are within three words of each other.
* captures alternative word endings

Additional file 1b EBSCO (CINAHL and SPORTDiscus) search strategy.

**Search Terms**
**1.** (physical* activ*)
**2.** (leisure N3 time)
**3.** (sport*)
**4.** (exercise)
**5.** (walk*)
**6.** (recreational)
**7.** (father* N3 (occupation* or education*))
**8.** (mother* N3 (occupation* or education*))
**9.** (parent* N3 (occupation* or education*))
**10.** (father* N3 (income or manual))
**11.** (mother* N3 (income or manual))
**12.** (parent* N3 (income or manual))
**13.** (father* N3 (social class or social status))
**14.** (mother* N3 (social class or social status))
**15.** (parent* N3 (social class or social status))
**16.** (child* N3 (social class or social status))
**17.** (early-life N3 (social class or social status))
**18.** (adolescen* N3 (social class or social status))
**19.** (father* N3 (socioeconomic or socio-economic))
**20.** (mother* N3 (socioeconomic or socio-economic))
**21.** (parent* N3 (socioeconomic or socio-economic))
**22.** (child* N3 (socioeconomic or socio-economic))
**23.** (adolescen* N3 (socioeconomic or socio-economic))
**24.** (early N3 (socioeconomic or socio-economic))
**25.** (early-life N3 (socioeconomic or socio-economic))
**26.** (child* N3 (deprivation or poverty))
**27.** (early-life N3 (deprivation or poverty))
**28.** (adolescen* N3 (deprivation or poverty))
**29.** (child* N3 overcrowding)
**30.** (adult*)
**31.** (midlife or mid-life)
**32.** (old*)
**33.** (later-life)
**34.** 1 OR 2 OR 3 OR 4 OR 5 OR 6
**35.** 7 OR 8 OR 9 OR 10 OR 11 OR 12 OR 13 OR 14 OR 15 OR 16 OR 17 OR 18 OR 19 OR 20 OR 21 OR 22 OR 23 OR 24 OR 25 OR 26 OR 27 OR 28 OR 29
**36.** 30 OR 31 OR 32 OR 33
**37.** 34 AND 35 AND 36
**38.** Remove duplicates from 37

N3 locates results where search terms are within three words of each other.
* captures alternative word endings
