# Supplementary material for: Childhood socioeconomic position and adult leisure-time physical activity: a systematic review
Source: Int J Behav Nutr Phys Act. 2015 Jul 3;12:92. doi: 10.1186/s12966-015-0250-0 (PMC4501082; doi:10.1186/s12966-015-0250-0)
Supplement: Additional file 2: — Data extraction form. [file 12966_2015_250_MOESM2_ESM.pdf]

Additional file 2 Data extraction form.

A. Reference details								
A1. Ref ID, 1st author, title, publication year,								
A2. Assessor's name and date of assessment								
B. Study details								
B1. Name of study/cohort								
B2. Design	Prospective cohort		Retrospective cohort		Case-control		Other	
B2A. If other:								
B3. Country, setting								
C. Childhood socioeconomic position								
C1. Parental occupation	Yes		No					
C2. Parental education	Yes		No					
C3. Other measures (list):								
C4. How ascertained	Prospectively		Retrospectively					
C5. Age recorded								
C6. Age referred to								
D. Physical activity (PA) outcomes								
D1. Parameters measured	Frequency		Type		Duration		Intensity	
D2. Type of leisure-time PA (LTPA) measured	Sport/ exercise		Gardening/ DIY		Total LTPA		Other	
D2A. If other please describe								
D3. How ascertained	Self-reported		Objective methods					
D4. Age ascertained								
D5. Variable details	Binary		Ordinal		Continuous		Other	
D5A. If other:								
E. Available participant numbers								
E1. Baseline	Yes		No		If yes, number			
E2. Excluded	Yes		No		If yes, number			
E3. Refused	Yes		No		If yes, number			
E4. Lost to follow-up	Yes		No		If yes, number			
E5. Other losses	Yes		No		If yes, number			
E6. Included in analysis	Yes		No		If yes, number			
E7. All accounted for	Yes		No					
F. Analysis								
F1. How results analysed	Descriptive/ Trend		Logistic regression		Linear regression		Other	
F1A. If other:								
F2. Included in analysis	Men and women		Men only		Women only			
F3. Only significant results presented	Yes		No					
G. Summary of results								
G1. Prevalence/Mean difference	Yes		No					
G2. Odds/Risk ratios	Yes		No					
G3. Regression coefficients	Yes		No					
G4. Confidence intervals (CIs)/ P-value/standard errors (SE)	Yes		No					
G5. Other	Yes		No					
G5A. If other								
H. References for screening								
H1. Reference numbers								

I. Effect estimates

Association tested	Number analysed	Type of effect estimate and category comparison/value of unit change	Effect estimate	95% CI; SE; p-value	Confounders included in analysis
1.					
2.					
