# Supplementary material for: Childhood socioeconomic position and adult leisure-time physical activity: a systematic review
Source: Int J Behav Nutr Phys Act. 2015 Jul 3;12:92. doi: 10.1186/s12966-015-0250-0 (PMC4501082; doi:10.1186/s12966-015-0250-0)
Supplement: Additional file 3: — Quality assessment form. [file 12966_2015_250_MOESM3_ESM.pdf]

Additional file 3 Amended Newcastle-Ottawa Quality Assessment Scale.

Note: A study can be awarded a maximum of two stars for each numbered item – (except number 5).

A. Selection

1) Representativeness of the exposed cohort
   a) Truly representative of the source population. ✵ ✵
   b) Somewhat representative of the source population. ✵
   c) Selected group of users e.g. nurses, volunteers.
   d) No description of the derivation of the cohort.

2) Ascertainment of childhood socioeconomic position (SEP)
   a) Prospectively from parents/participants when aged ≤18 years. ✵✵
   b) Retrospectively collected with attempts to reduce recall bias (e.g. life-grid and structured interview techniques). ✵
   c) Retrospectively collected without attempts to reduce recall bias.
   d) No description.

B. Comparability

3) Comparability of cohorts on the basis of the design/analysis
   a) Study controls for adult SEP. ✵
   b) Study controls for any additional relevant factors (e.g. age, sex). ✵
   c) Only unadjusted model presented.

C. Outcome

4) Assessment of physical activity
   a) Objective methods (heart-rate monitoring/accelerometer). ✵ ✵
   b) Self-reported using validated questionnaire/diary/interview. ✵
   c) Self-report.
   d) No description.

5) Adequacy of cohort follow-up
   a) Complete follow up - all subjects accounted for. ✵
   b) Subjects lost to follow up unlikely to introduce bias (≥75% follow-up or description provided of those lost). ✵
   c) <75% follow-up and no description of those lost.
   d) No statement.
